# Capacitive Heart-Rate Sensing on Touch Screen Panel with Laterally Interspaced Electrodes

**DOI:** 10.3390/s20143986

**Published:** 2020-07-17

**Authors:** Junhyung Kim, Wonho Song, Sungchul Jung, Yuna Kim, Wonsang Park, Bonghyun You, Kibog Park

**Affiliations:** 1 School of Electrical and Computer Engineering, Ulsan National Institute of Science and Technology (UNIST), Ulsan 44919, Korea; junhyung@unist.ac.kr; 2 Department of Physics, Ulsan National Institute of Science and Technology (UNIST), Ulsan 44919, Korea; iiu13456@unist.ac.kr; 3 SK hynix, Icheon-si, Gyeonggi-do 17336, Korea; sinky0623@unist.ac.kr; 4 Samsung Display Giheung Campus, Yongin-si, Gyeonggi-do 17113, Korea; yuna0731.kim@samsung.com (Y.K.); wonsang.park@samsung.com (W.P.); yubong@samsung.com (B.Y.)

**Keywords:** heart-rate sensing, capacitive touch screen panel, laterally-interspaced electrodes, ulnar artery, effective dielectric constant, fast fourier transformation, biomedical monitoring, multi-functional sensor

## Abstract

It is demonstrated that the heart-rate can be sensed capacitively on a touch screen panel (TSP) together with touch signals. The existing heart-rate sensing systems measure blood pulses by tracing the amount of light reflected from blood vessels during a number of cardiac cycles. This type of sensing system requires a considerable amount of power and space to be implemented in multi-functional mobile devices such as smart phones. It is found that the variation of the effective dielectric constant of finger stemming from the difference of systolic and diastolic blood flows can be measured with laterally interspaced top electrodes of TSP. The spacing between a pair of non-adjacent top electrodes turns out to be wide enough to distinguish heart-rate signals from noises. With the aid of fast Fourier transform, the heart-rate can be extracted reliably, which matches with the one obtained by actually counting heart beats on the wrist.

## 1. Introduction

There has been a great deal of interest in integrating bionic sensors into mobile devices including fingerprint [1,2,3], skin moisture [4,5], blood glucose level [6], blood pressure [7], and heart-rate [8,9,10,11,12]. Mobile bionic sensors have made significant advances in biometric authentication and health monitoring. In terms of accessibility, touch screen panels (TSPs) are particularly advantageous for implementing bionic sensing functionalities, although their actual embedment is quite challenging [13,14]. The on-screen fingerprint sensor is one successful example of such a TSP-mounted bionic sensor [3]. Especially, a considerable number of attempts for implementing heart-rate sensors into mobile devices including smart phones, smart watches, and other wearable devices have been made in recent years [8,9,11,12,15,16,17,18]. Portable heart-rate sensors are extremely helpful to perceive and prevent cardiac disorders like arrhythmia [19,20]. The commercialized heart-rate sensors mostly rely on the optical measurements called photoplethysmography, which exploits the difference in the amount of reflected light between systolic and diastolic phase of blood vessel [8,15,21]. Therefore, they require both light transmitter and receiver, which necessitate a substantial amount of power and a dedicated space to be implemented. An alternative way is the electrocardiogram (ECG) that can detect the electrical charges induced by cardiac cycles [9,17,22,23]. The ECG requires multiple electrical contacts onto human bodies, making the system somewhat complex and bulky.

In this work, it is demonstrated experimentally that heart-rate can be sensed by analyzing the time-dependent capacitive signals when a particular portion of finger touches the TSP surface. While the finger touches the surface of the TSP constructed with two vertically-spaced layers of crossing electrodes, some amount of the electric field lines starting from the upper (or top) electrode acting as transmitter (T_X_) is absorbed by the finger. Then, the mutual capacitance between the upper electrode and the lower (or bottom) electrode acting as receiver (R_X_) is reduced so that the finger touch can be sensed [14,24]. Here, the amount of absorbed electric field lines is directly related to the effective dielectric constant of finger which is modulated regularly by the periodic behavior of blood flow during cardiac cycles. When choosing a pair of top and bottom electrodes as T_X_ and R_X_, the oscillatory change of measured capacitance reflecting the modulation of finger dielectric constant is as small as comparable to the typical noises occurring during measurements. Even in this case, the capacitance oscillation caused by cardiac cycles can still be observed. However, the measured capacitance is affected mostly by the medium between top and bottom electrodes and thus the heart-rate extracted from the capacitance oscillation will be non-reliable [19,25]. On the other hand, if two interspaced top electrodes are used as T_X_ and R_X_, the capacitance oscillation associated with the modulation of finger dielectric constant can be magnified much more. It is observed that the capacitance oscillation can be enhanced enough to distinguish the heart beat signals reliably from the noises with a proper separation of two interspaced electrodes. Quite interestingly, a convenient method to achieve the proper separation while keeping the high spatial resolution for touch sensing is found to be utilizing non-adjacent interspaced electrodes as T_X_ and R_X_. This simple method will be quite efficient for resolving the issue of the capacitance oscillation being reduced with a protective layer positioned on top of the top electrodes, which is a mandatory component in a commercial TSP.

## 2. Materials and Methods

### 2.1. Fabrication of Capacitive Touch Screen Panel

**Figure 1:**
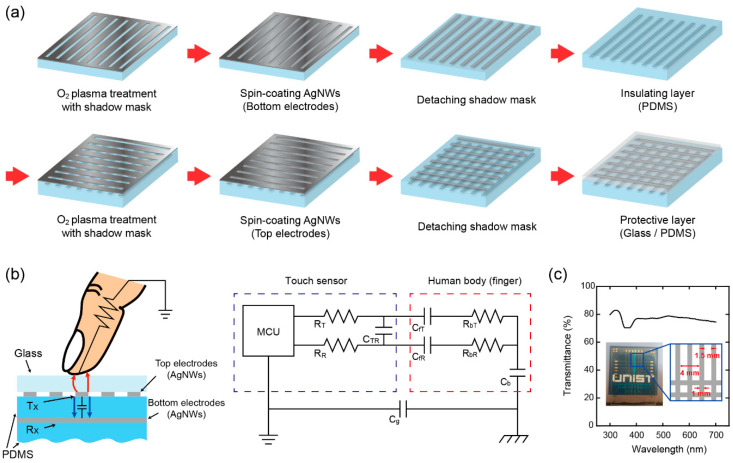
(**a**) Non-lithographic processes of fabricating touch screen panel (TSP). (**b**) Schematic view of the operational principle of fabricated TSP and its equivalent circuit. (**c**) The photo of fabricated TSP and its transmittance at the crossing area of top and bottom electrodes. The fabricated TSP has 6 × 6 crossed lines of top and bottom electrodes. The width of electrode (both top and bottom) is 1 mm and the spacing between electrodes is 1.5 mm.

The non-lithographic fabrication procedures of capacitive TSP [13,26] are shown in Figure 1a. First, a polydimethylsiloxane (PDMS) film is spin-coated on a Si wafer at 200 rpm for 60 sec and baked in an oven at 100 °C for 20 min to make a ~300 μm thick soft substrate. After being detached from the Si wafer, the surface of PDMS substrate is then treated selectively with O_2_ plasma while being covered by a pre-patterned shadow mask. The O_2_ plasma treatment is performed with the power of 100 W and the gas flow of 50 sccm for 5 min. The shadow mask has line openings of ~1 mm width through which the PDMS surface is exposed to the O_2_ plasma. With this process, the PDMS surface exposed to O_2_ plasma is converted from hydrophobic to hydrophilic. Then, silver nanowires (AgNWs) dissolved in deionized (DI) water will be adhered selectively only on the converted (hydrophilic) area of PDMS surface so that AgNW electrode patterns can be formed without any lithography processes. On the PDMS surface treated with O_2_ plasma, an AgNW solution film is spin-coated at 300 rpm for 15 sec and baked on a hot plate at 105 °C for 10 min to dry off water and form the bottom electrode lines. On top of the AgNW bottom electrode lines, another PDMS film is spin-coated by following the procedures identical to those utilized for the PDMS substrate. This PDMS film takes a role of an insulating layer separating top and bottom electrodes. As performed previously for the PDMS substrate, the surface of PDMS insulating layer is exposed to O_2_ plasma while being covered with the shadow mask to induce line-shape hydrophilic regions. Then, the top electrode lines are formed on the hydrophilic regions by spin-coating an AgNW solution film and baking it in the same way as for the bottom electrode lines. Finally, a protective layer, either glass or PDMS film, can be attached for preventing potential physical damages given to the AgNW top electrode during repeating finger touch.

Figure 1b shows the working mechanism of touch sensing in the fabricated capacitive TSP. The red lines indicate the electric field loss to the finger and the blue lines show the electric field between top and bottom electrodes. Due to the electric field loss to the touching finger, the mutual capacitance between top and bottom electrodes is measured to decrease. The equivalent circuit corresponding to the finger touch is also shown in Figure 1b where the C*_fT_* (C*_fR_*) represents the additional capacitance between finger and transmitter (receiver) introduced by the finger touch. Figure 1c shows the transmittance of fabricated TSP in the visible range, ~78% at 550 nm in particular.

### 2.2. Periodically-Varying Effective Dielectric Constant of Finger Synchronized to Cardiac Cycles

The ulnar artery is a blood vessel distributed in the palms and fingers. For a period of heart beat, the volume flow rate of blood in the ulnar artery increases about 3.5 times for the systolic phase compared with that of the diastolic phase. The dielectric constant of blood (ε_blood_ ≈ 10^4^) is quite large, more than a hundred times that of water (ε_water_ ≈ 80) at the operating frequency of commercial TSP, 200 kHz [27]. Therefore, although the volume occupied by blood vessels in the finger is insignificant, a considerable amount of change in the effective dielectric constant of finger can arise by heart beat [19,20,28].

### 2.3. Fringing Electric Field Between Laterally Interspaced Top Electrodes

The heart-rate sensing in our TSP relies on the fringing electric field between two laterally interspaced top electrodes, similarly to interdigitized capacitive skin moisture sensor so-called corneometer. In the corneometer, the capacitance between two interdigitized electrodes is modulated by the amount of the skin moisture in the stratum corneum. The penetration depth of the electric field into the touching finger increases as the spacing between the interdigitized electrodes increases. Conventional skin moisture sensors use the spacing of several micrometers to make the fringing electric field reach optimally the stratum corneum, which is distributed in ~10 μm depth from the surface of skin. In case of the heart-rate sensing, the spacing between two laterally interspaced electrodes needs to increase to several millimeters so that the fringing electric field can reach the blood vessel residing further deeper from the skin surface than the stratum corneum.

## 3. Results and Discussion

### 3.1. Touch Signal Sensing with Top and Bottom Electrodes

**Figure 2:**
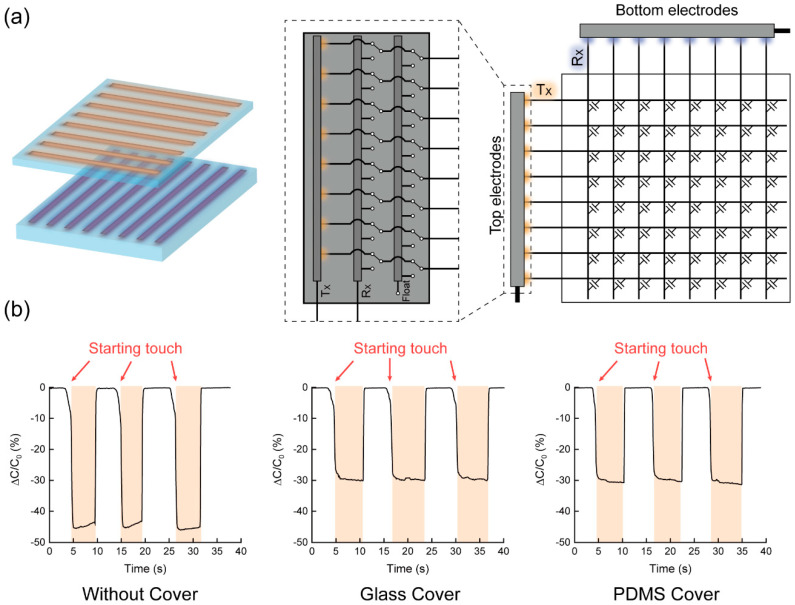
(**a**) Schematic view of electrode/circuit configuration of TSP for touch sensing mode and (**b**) capacitance change with finger touch for the three different cases of cover layer (no, glass, polydimethylsiloxane (PDMS)).

When operating the fabricated TSP for touch sensing, the top and bottom electrodes were used as T_X_ and R_X_ as shown in Figure 2a. This is identical to the working mechanism of the conventional TSP as described previously. Figure 2b shows the mutual capacitance measured between top and bottom electrodes while the continuous touching and untouching of finger occurred. With the finger touching, the mutual capacitance decreased by ~46% with respect to its value without the finger touching. The finger works as a grounded electrode and the electric field starting from the T_X_ line is partly absorbed by the finger. With the protective layer including ~500 μm of glass or ~300 μm of PDMS film, the reduction of the capacitance dwindled down to ~30%. This is due to the increased distance between the finger and the T_X_ electrode which corresponds to the decrease of the effective capacitance between them. The capacitance measurements were performed by using E4980A precision LCR meter (Keysight Technologies, Santa Rosa, CA, USA).

One thing to note here is that a switch circuit shown in the dotted square of Figure 2a is necessary to select the functionality of each top electrode among T_X_, R_X_, or floating line. In order to perform the heart-rate measurement properly, it is needed to know where the finger has touched. Therefore, all the top electrodes should function as T_X_ lines with the bottom electrodes acting as R_X_ lines for touch sensing in the first place. Once the location of finger touch is identified, a pair of interspaced top electrodes in the finger-touched region are used for sensing heart-rate. In this case, one of the top electrode pair should be used as T_X_ line and the other as R_X_ line while all the other top electrodes are floated electrically. Hence, it is essential to change the configuration of signal transmission and reception through the top electrodes as needed for the on-panel heart-rate measurement with TSP. In case that the touching finger covers several pairs of top and bottom electrodes and multiple touch signals are detected, a pair of top and bottom electrodes giving the largest touch signal will be identified to locate the touch center. Once the touch center is identified, two interspaced top electrodes at the touch center will be chosen to measure the heart-rate.

### 3.2. Heart-Rate Extracted from the Time Trend of Capacitance

**Figure 3:**
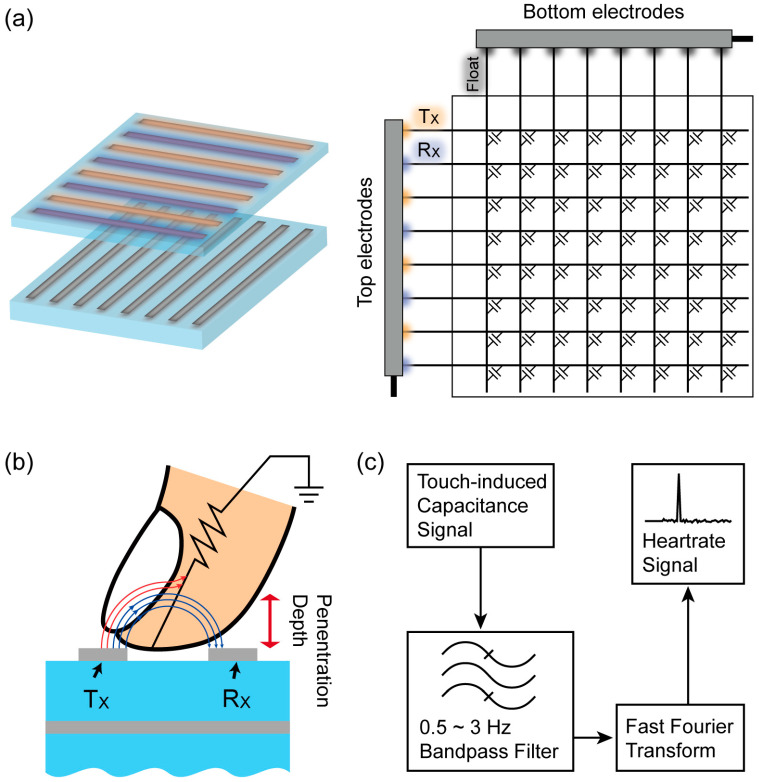
Schematic view of (**a**) electrode/circuit configuration and (**b**) the operational principle of TSP for heart-rate sensing mode. (**c**) Schematic diagram of signal processing flow to obtain the frequency spectrum of measured capacitance.

As mentioned above, two interspaced top electrodes can be used as T_X_ and R_X_ in the heart-rate sensing mode (Figure 3a). The gap between the two electrodes was ~3 mm so that a considerable amount of electric field lines between them could reach the branches of radial or ulnar arteries in the finger. Although the dielectric constant of blood vessel is enormously large and the gap between the two top electrodes is chosen optimally to have the electric field lines reach the blood vessel, the modulation of measured capacitance due to heart beats is found to be rather small and a certain degree of post signal processing is required to make it distinct from the noises arising during measurements. The post signal processing procedures adopted here are shown in Figure 3c, which were performed with the built-in functionalities of the commercial data analysis package, Origin (OriginLab Corp., Northampton, MA, USA).

**Figure 4:**
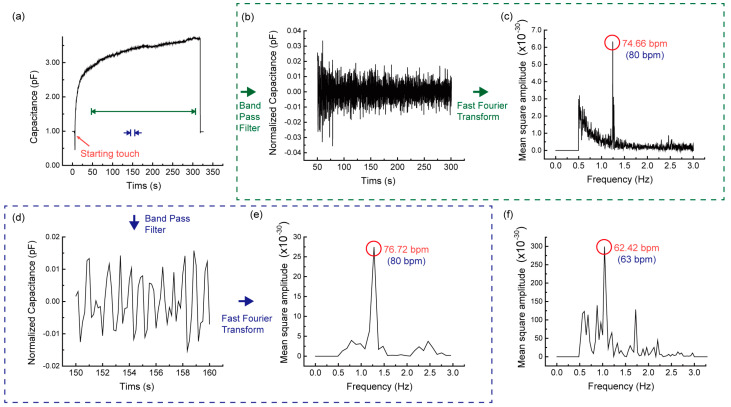
(**a**) The time trend of capacitance measured for heart-rate sensing without cover layer. The green dotted-line encloses the signal processing results for the oscillatory part of measured capacitance within the time interval of ~250 sec and the blue dotted-line encloses the corresponding results for the time interval of ~10 sec. (**b**,**d**) show the normalized capacitance after going through band pass filtering (0.5–3.0 Hz) and (**c**,**e**) show the frequency spectrum obtained with the subsequent fast Fourier transformation (FFT), (**f**) is the frequency spectrum of the capacitance signal measured for a different person which results from the same signal processing procedures.

The capacitance data measured with the heart-rate sensing configuration is shown in Figure 4a. The capacitance is found to increase ~200% when the finger touches directly the top electrodes of TSP without any protective layer. The increase of measured capacitance is understandable because the dielectric material between the two top electrodes is replaced from the air to the finger with a dielectric constant higher than the air. It is apparent that a regular oscillation with its amplitude of ~40 fF exists in the capacitance measured during finger touch. We have performed the post signal processing for two different time intervals to evaluate the sampling dependence of extracting heart-rate values. Figure 4b,c show the oscillatory part of measured capacitance covering the time interval of ~250 sec and its frequency spectrum, which was obtained by going through band pass filtering (0.5–3.0 Hz) and fast Fourier transformation (FFT) sequentially. Here, it is noted that the band pass range of 0.5–3.0 Hz amounts to the typical range of the human heart-rate, 30–180 bpm (beat per minute). In the frequency spectrum, a sharp peak is observed at the frequency of 1.24 Hz corresponding to the heart-rate of 74.66 bpm. As a comparison, the heart-rate was estimated to be ~80 bpm when heart beats were counted directly on the wrist. For the shorter time interval of ~10 sec shown in Figure 4d,e, the heart-rate was obtained to be 76.72 bpm from the frequency spectrum which is very close to the heart-rate for the longer time interval. The similarity of the extracted heart-rate between the two time intervals ensures that the oscillating feature in the measured capacitance is quite regular and robust. This insensitivity of extracted heart-rate to the sampling size allows reducing the measurement time down to ~10 sec, which will be quite advantageous in implementing the capacitive heart-rate sensing functionality in TSP. In order to evaluate the reliability of extracting heart-rate from capacitance measurement, the heart-rate of another person was also measured. This time, the peak in the frequency spectrum was observed at 62.42 bpm and the corresponding direct-counting on the wrist was found to be ~63 bpm, almost identical to each other. The oscillation amplitude of heart beat signals can vary depending on person. Then, the corresponding amplitude in the frequency domain will vary accordingly. In addition, the oscillation amplitude can also be affected by the touching force since the TSP consists of soft PDMS layers and the finger is somewhat soft. However, the amplitude change caused by the touching force doesn’t alter the oscillating characteristics of heart beat signals and thus the oscillation frequency can be extracted reliably after going through the band pass filtering and FFT processes.

### 3.3. Extraction of Heart-Rate with a Protective Layer Covering High-Resolution Top Electrodes

**Figure 5:**
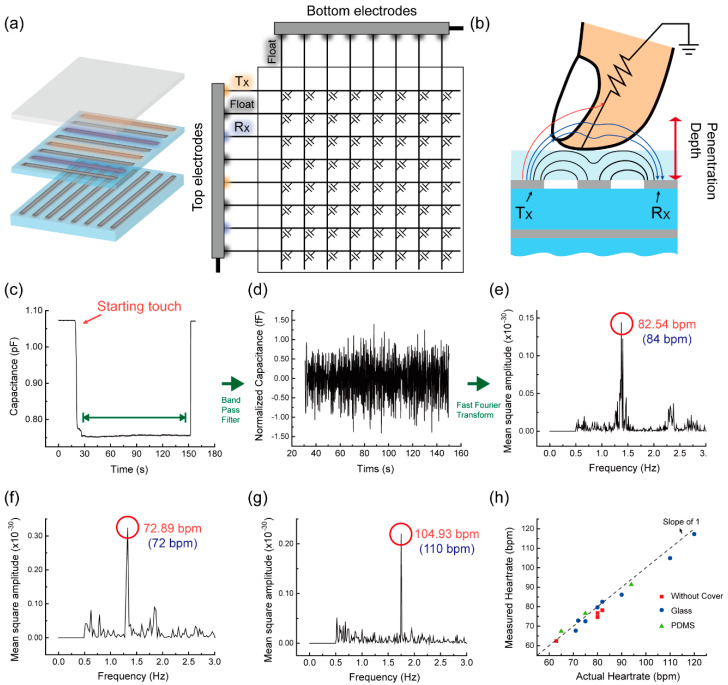
Schematic view of (**a**) electrode/circuit configuration and (**b**) the operational principle of TSP for heart-rate sensing with cover glass. The red lines represent the electric field lines lost to the finger and the blue lines indicate the electric field lines going through the finger. (**c**) Capacitance change when a finger touches the surface of cover glass, (**d**) normalized capacitance after going through band pass filtering (0.5–3.0 Hz), and (**e**) frequency spectrum obtained from FFT. Frequency spectra of the capacitance data measured (**f**) for another person and (**g**) after exercise. The values inside the parentheses of (**e**–**g**) are the heart-rates counted directly on the wrist. (**h**) Correlation between capacitively-measured and directly-counted heart-rates for no cover layer, glass cover layer, and PDMS cover layer.

For a commercial TSP, a protective layer covering top electrodes is required typically to prevent physical and chemical damages from being given to them. In this case, the distance between finger blood vessels and top electrodes will increase so that the electric field lines between two nearest interspaced (adjacent) top electrodes can hardly reach the blood vessels. One straightforward way to resolve this penetration depth issue is to enlarge the gap between T_X_ and R_X_. However, the simple enlargement of the interspacing between adjacent top electrodes will lower the spatial resolution of touch sensing, which is not desirable in a commercial TSP. One efficient way to increase the gap between T_X_ and R_X_ electrodes for heart-rate sensing without influencing the spatial resolution of touch sensing is to use two non-adjacent electrodes. Figure 5a shows such an example where a pair of next nearest-neighbor top electrodes are used as T_X_ and R_X_ and the electrode between them is floating. The distribution of electric field lines when a finger touches on the ~0.5 μm thick glass protective layer of TSP is shown in Figure 5b.

Figure 5c shows the mutual capacitance signal measured between the non-adjacent T_X_ and R_X_. Here, the measured capacitance was found to decrease overall with finger touch, differently from the adjacent T_X_ and R_X_ case with no protective layer (Figure 4a). Since the dielectric constant of glass is ~5 times larger than that of air, the effective dielectric constant between T_X_ and R_X_ is already pretty large. Hence, the relative increment of effective dielectric constant due to the finger will be smaller than the case of no protective layer. In this case, the electric field loss to the finger can be more dominating and lead to the capacitance decrease. Nonetheless, the clear oscillation in the measured capacitance was still observed, indicating the cyclic modulation of the dielectric constant of finger synchronized with heart beats. As done previously, the 0.5–3.0 Hz of band pass filtering (Figure 5d) and FFT (Figure 5e) were performed. The oscillation amplitude was estimated to be ~3 fF. In the frequency spectrum, a clear peak was observed at ~82.54 bpm while the heart-rate counted on the wrist was 84 bpm. The heart-rate was measured under several different circumstances including multiple persons and their breathing condition (before and after physical exercise). Figure 5f,g are such examples where the closeness of the heart-rate from the capacitance measurements and that from the direct counting on the wrist can be seen clearly again. As an alternative protective layer, we also tested a ~300-μm-thick PDMS layer and the heart-rate extracted from capacitance measurements matched well with the directly-counted one. More details associated with the PDMS protective layer can be found in Supplementary Materials (Figure S1). Figure 5h shows that the correlation between capacitively-measured and directly-counted heart-rate, covering all three cases of no protective layer, glass protective layer, and PDMS protective layer, almost approaches the ideal case (slope of 1). This implies that the procedures presented in our work can be an efficient and reliable solution to measure the heart-rate with a conventional capacitive type TSP.

## 4. Conclusions

In summary, we demonstrated experimentally that the heart-rate can be sensed on a conventional capacitive type TSP by tracing the time-dependent capacitance between two laterally interspaced top electrodes with a proper spacing. The cyclic change of the effective dielectric constant of finger due to the periodic blood flow synchronized with cardiac cycles was found to produce the regular oscillatory pattern in the measured capacitance. With the aid of band pass filtering and FFT, the heart-rate can be extracted reliably, matching with the one obtained by actually counting heart beats on the wrist. Our work can provide a readily-applicable way for implementing the on-panel heart-rate sensing functionality into the conventional TSP without deteriorating the spatial resolution of touch sensing, which extends the usage of TSP to the real-time health monitoring.

## Supplementary Materials

The following are available online at https://www.mdpi.com/1424-8220/20/14/3986/s1, Figure S1: In case of the PDMS protective layer, the capacitance signal when a finger touches the PDMS surface, normalized capacitance signal after band pass filtering, and its frequency spectrum obtained from FFT corresponding to the directly-counted heart-rate of (a) 75 bpm and (b) 94 bpm.
