# Supplementary material for: Capacitive Heart-Rate Sensing on Touch Screen Panel with Laterally Interspaced Electrodes
Source: Sensors (Basel). 2020 Jul 17;20(14):3986. doi: 10.3390/s20143986 (PMC7412254; doi:10.3390/s20143986)
Supplement: Supplementary file 1 [file sensors-20-03986-s001.pdf]

Supplementary Information

Capacitive Heart-Rate Sensing on Touch Screen Panel with Laterally Interspaced Electrodes

Junhyung Kim [1], Wonho Song [2], Sungchul Jung [3], Yuna Kim [4], Wonsang Park [4], Bonghyun You [4], and Kibog Park [1,2,*]

[1] School of Electrical and Computer Engineering, Ulsan National Institute of Science and Technology (UNIST), Ulsan 44919, Republic of Korea; junhyung@unist.ac.kr

[2] Department of Physics, Ulsan National Institute of Science and Technology (UNIST), Ulsan 44919, Republic of Korea; iiu13456@unist.ac.kr

[3] SK hynix, Icheon 17336, Republic of Korea; sungchul.jung@sk.com

[4] Samsung Display Giheung Campus, Yongin-si, Gyeonggi-do, 17113, Republic of Korea; yuna0731.kim@samsung.com (Y. K.); wongsang.park@samsung.com (W. P.); yubong@samsung.com (B.Y.)

* Correspondence: kibogpark@unist.ac.kr; Tel.: +(82)-052-217-2111

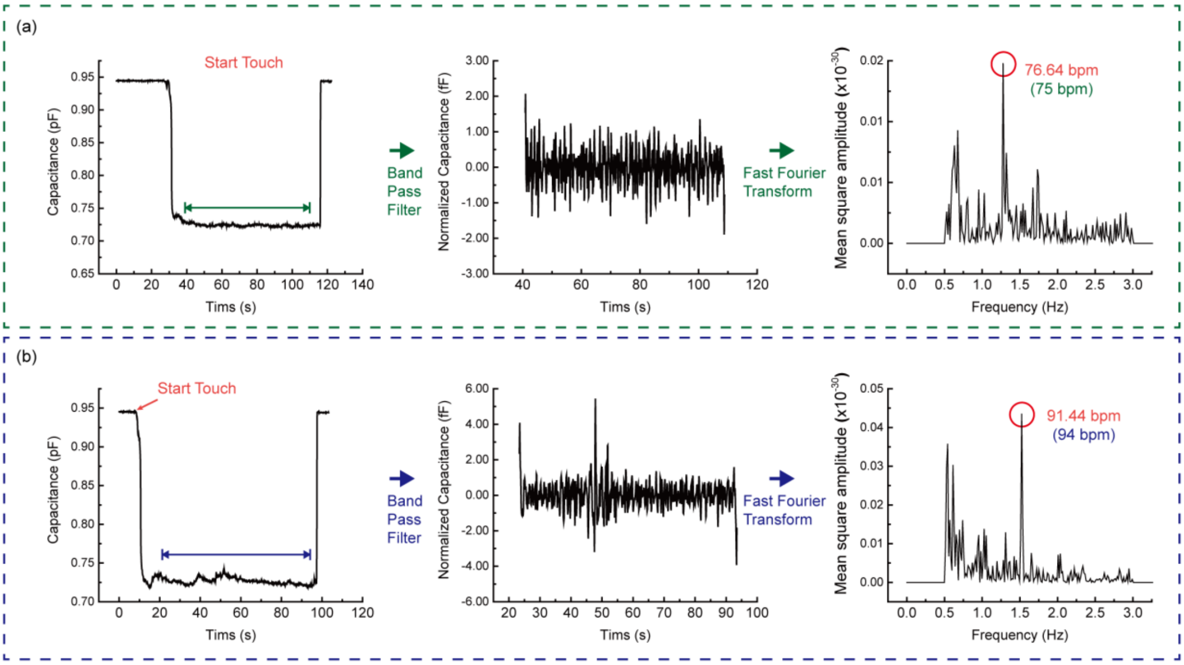

Figure S1. In case of the PDMS protective layer, the capacitance signal when a finger touches the PDMS surface, normalized capacitance signal after band pass filtering, and its frequency spectrum obtained from FFT corresponding to the directly-counted heart-rate of (a) 75 bpm and (b) 94 bpm
